# From Molecular Recognition to Clinical Readout: Design Principles for Functional Nucleic Acid–Material Biosensors in Medical Diagnostics

**DOI:** 10.3390/molecules31183358

**Published:** 2026-09-21

**Authors:** Qiming Chen, Chengtian Xue, Yuanlong Hu, Qiao Hong, Zhanmin Liu

**Affiliations:** School of Life Sciences, Shanghai University, Shanghai 200444, China

**Keywords:** functional nucleic acids, aptamers, DNAzymes, CRISPR diagnostics, nucleic-acid amplification, nanomaterials, clinical translation, point-of-care testing, sample-to-answer

## Abstract

Functional nucleic acids can connect molecular recognition with chemical signal generation, but their diagnostic value depends on the performance of the complete sample-to-answer pathway. This critical narrative review examines representative studies published through 31 July 2026, with emphasis on recognition, amplification, material interfaces, sample preparation, readout and clinical interpretation under realistic conditions. Hybridization and ligation probes, aptamers, DNAzymes, DNA nanostructures and CRISPR-associated systems are compared by specificity, kinetics, leakage and matrix compatibility. Rolling circle amplification, hybridization chain reaction, catalytic hairpin assembly and enzymatic isothermal amplification are evaluated as reaction networks whose products must remain accessible to the selected interface. Functional materials are classified by their actual analytical role, including transduction, signal amplification, capture/enrichment, spatial organization and reagent storage. Evidence is distinguished between mechanistic studies, spiked matrices, clinical specimens, manufactured-format reproducibility and demonstrated clinical utility. We further integrate sample-to-answer workflow, assay time, complexity, regulatory considerations and clinically relevant decision thresholds. Across the literature, reliable performance depends on selective recognition before high-gain reactions, compatibility between amplification products and interfaces, explicit controls for inhibition and leakage, and validation across independent lots and representative clinical populations. These principles define a path from analytical proof of concept to reproducible and clinically interpretable diagnostic testing.

## 1. Introduction

Functional nucleic acids can connect molecular recognition with chemical signal generation, although their value in medical diagnostics depends on the performance of the complete assay. This critical narrative review examines representative studies published through 31 July 2026, with particular attention to the interactions among recognition, amplification, material interfaces and readout under realistic sample conditions. Hybridization and ligation probes, aptamers, DNAzymes, DNA nanostructures and CRISPR-associated systems are discussed in terms of specificity, reaction kinetics, background leakage and matrix compatibility. Rolling circle amplification, hybridization chain reaction, catalytic hairpin assembly and enzymatic isothermal amplification are considered as reaction networks whose products must remain accessible to the selected interface. Noble-metal nanostructures, fluorescent nanoclusters, carbon and two-dimensional materials, metal–organic frameworks, nanozymes, magnetic particles, hydrogels, paper and time-resolved labels are evaluated according to their functions in signal generation, target enrichment, reagent stabilization and interference control. The available evidence is considered at five levels, ranging from mechanistic studies in buffer to clinical utility. Across the reported platforms, reliable performance depends on placing selective recognition before high-gain reactions, maintaining compatibility between amplification products and material interfaces, and including controls that distinguish inhibition, leakage and false-positive pathways. These considerations define the conditions under which functional nucleic acid–material systems can progress from analytical demonstrations to reproducible diagnostic tests.

Medical diagnostics must transform a molecular measurement into an actionable result despite biological heterogeneity, complex matrices, pre-analytical variation and constraints on time, equipment and operator expertise. Functional nucleic acids are particularly attractive because the same programmable polymer can provide sequence recognition, ligand binding, catalysis, nanoscale organization or participation in an amplification reaction. This versatility supports assays for nucleic acids, proteins, metabolites, pathogens and cells, while allowing molecular recognition to be coupled directly to optical, electrochemical or material-assisted signal generation.

The central problem is that performance gains at one stage can create failure modes elsewhere. Probe affinity measured in purified buffer may decrease in saliva, serum, urine or stool because of nuclease degradation, competitive adsorption, electrostatic screening or altered target transport. Amplification can increase signal while also increasing leakage, aerosol contamination, reagent burden and assay time. Materials can increase surface area or catalytic activity while simultaneously increasing nonspecific adsorption, lot variation or transport barriers. We therefore use “clinically interpretable readout” to mean a result produced by a predefined analytical and interpretive rule that is sufficiently specific, reproducible and calibrated in the intended specimen to support the stated clinical purpose; it does not mean that any analytically measurable signal is clinically useful.

Most previous reviews organize functional nucleic-acid biosensors primarily by receptor, material, detector or application. Recent reviews have provided valuable depth in specific dimensions, including nucleic-acid electrochemical sensors, nucleic-acid point-of-care testing, CRISPR/Cas12 engineering, and CRISPR signal-transduction strategies [1,2,3,4,5,6,7,8]. These studies demonstrate the maturity of individual technology classes, but they generally do not integrate, in one operational framework, molecular recognition, sample preparation, amplification, material function, interface compatibility, assay complexity, time-to-result, manufacturing variation and the evidence required for clinical interpretation. Our contribution is therefore not the invention of a new sensor class, but a cross-stage synthesis that makes dependencies between these dimensions explicit. In particular, we ask whether specificity is preserved after amplification, whether amplified products remain accessible to the chosen material interface, and whether the final signal is supported by appropriate controls and clinical evidence. The literature published through 31 July 2026 is considered, with recent studies prioritized for platform design and translation.

For this review, functional nucleic acids (FNAs) are defined operationally as DNA or RNA molecules whose analytical role extends beyond passive sequence storage to include specific recognition, conformational switching, catalysis, programmable strand exchange, target-gated reaction control or nanoscale organization. Conventional hybridization probes are included when they participate in such an engineered analytical function (for example, ligation gates or strand-displacement circuits), whereas routine PCR primers or passive capture oligonucleotides without a functional transition are not treated as FNA modules. This definition also clarifies the boundary between a molecular circuit and a biosensor: a circuit is considered part of the biosensor only when its output is coupled to a measurable transduction/readout and interpreted within a defined sample-to-answer workflow.

This molecular versatility does not, however, ensure satisfactory diagnostic performance. Probe affinity measured in purified buffer may be reduced by nuclease degradation, competitive adsorption, electrostatic screening or slow target transport in saliva, blood, urine or stool. Nanostructured materials can increase nominal surface area while also increasing nonspecific adsorption or restricting access to capture strands. Similarly, an amplification reaction with an attomolar detection limit may introduce leakage, aerosol contamination, long assay times or temperature requirements that are unsuitable for decentralized testing. The performance of the complete molecular-to-readout sequence is therefore more informative than the maximum signal obtained from any single recognition element or material.

**Figure 1:**
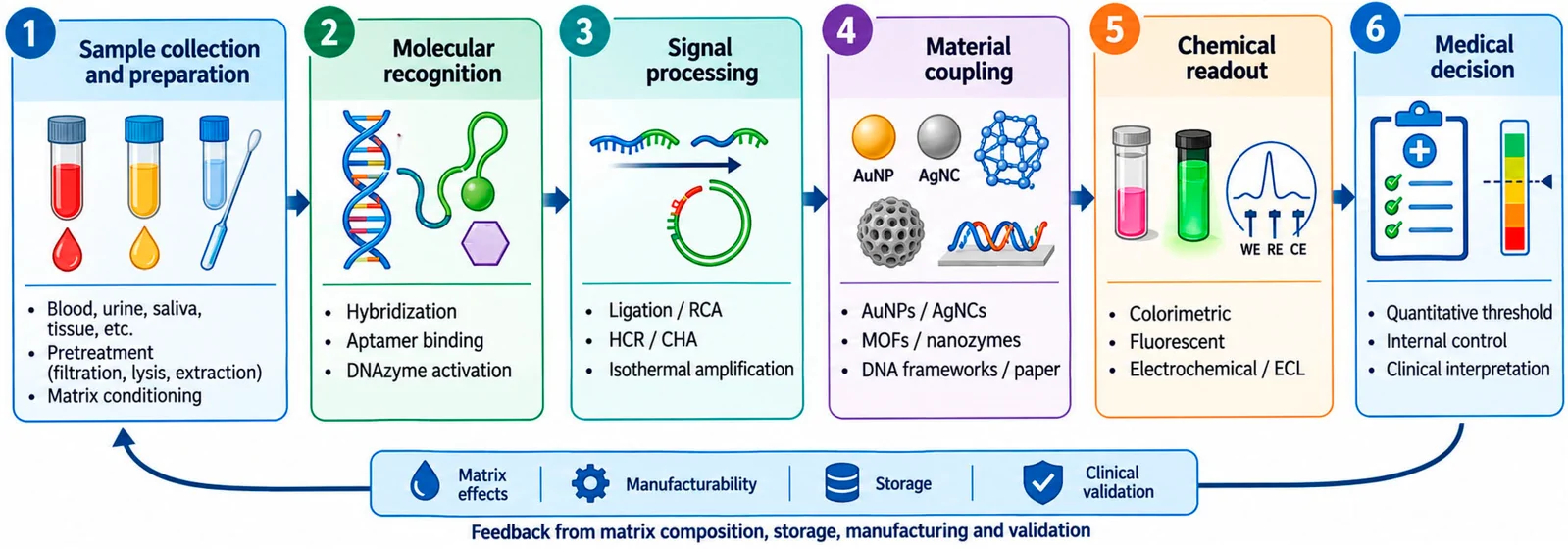
Sample-to-clinical organization of functional nucleic acid–material biosensors. The framework follows specimen collection and preparation, molecular recognition, target-dependent signal processing, material coupling, chemical readout and clinical interpretation, with feedback from matrix composition, storage, manufacturing and validation.

Most reviews classify functional nucleic-acid biosensors according to receptor type, material or detector. Although this approach is useful for describing individual technologies, it gives less attention to the way in which limitations at one stage affect the rest of the assay. In this review, the complete molecular-to-decision sequence is used as the basis for comparison. The literature published through 31 July 2026 is examined with emphasis on three recurring requirements: selective recognition before signal amplification, physical compatibility between reaction products and the material interface, and controls that reveal inhibition, leakage and common false-positive pathways. An evidence hierarchy and a minimum reporting checklist are also used to distinguish analytical proof of concept from diagnostic readiness. Figure 1 summarizes this molecular-to-clinical sequence, including the feedback imposed by matrix composition, storage, manufacturing and clinical validation, and provides the organizational basis for the sections that follow.

This article presents a critical narrative review rather than a systematic review or meta-analysis. To improve transparency, the literature identification strategy was explicitly documented around four source classes: PubMed/MEDLINE, Web of Science Core Collection, Scopus and Google Scholar, supplemented by backward and forward citation tracking. Core search concepts combined terms for functional nucleic acids (functional nucleic acid, aptamer, DNAzyme, DNA nanostructure, CRISPR/Cas), amplification (RCA, HCR, CHA, LAMP, RPA/RAA, isothermal amplification), materials (nanomaterial, nanoparticle, MOF, nanozyme, electrode, graphene, MXene, paper, hydrogel), readouts (electrochemical, fluorescence, colorimetric, SERS, lateral flow, FET) and translation (clinical, patient, specimen, serum, plasma, saliva, urine, matrix, point-of-care, reproducibility, stability, manufacturing). Searches were updated through 31 July 2026. Foundational reports were retained when required to define mechanisms; the 2023–2026 literature was preferentially used for contemporary comparisons and clinical translation.

Because the original iterative narrative search was not prospectively logged as a PRISMA-style systematic search, an exact retrospective count of all initially retrieved records cannot be reconstructed without inventing data. We therefore do not report a fabricated retrieval number. Instead, Supplementary Figure S1 records the documented identification and selection logic, the principal inclusion/exclusion criteria, and the limitation that this is a narrative rather than exhaustive systematic review. Studies were retained when they contributed evidence on recognition, amplification, material-interface constraints, sample preparation, readout, reproducibility or clinical translation. Preference was given to independent studies involving clinical specimens, reference methods, manufactured sensor batches, biofluid matrices or direct detector comparisons. Food- and veterinary-sensing studies were retained only when their architecture was directly relevant to medical diagnostics and their original application was explicitly labeled in the text and tables.

## 2. Scope and Conceptual Framework

### 2.1. Review Scope and Selection Rationale

This article presents a critical narrative review rather than a systematic review or meta-analysis. Relevant studies were identified through iterative keyword searches and backward and forward citation tracking. The search combined terms related to functional nucleic acids, aptamers, DNAzymes, DNA nanostructures, CRISPR, rolling circle amplification, hybridization chain reaction, catalytic hairpin assembly and isothermal amplification with terms describing biosensors, nanomaterials, electrochemical and optical detection, lateral flow, clinical specimens, matrix effects, stability and point-of-care testing. Foundational reports were included when needed to explain a mechanism, whereas the recent literature through 31 July 2026 was emphasized for platform design and translation. When available, preference was given to independent studies involving clinical specimens, reference methods, manufactured sensor batches, biofluid matrices or direct comparisons of detector formats.

Studies were retained when they provided information on a recognition mechanism, amplification reaction, material-interface constraint, readout process or barrier to clinical use. Mechanistically informative primary studies, clinically relevant specimen formats and recent reviews were given priority. Food- and veterinary-sensing studies were included only when their architectures were directly relevant to medical diagnostics, and their original applications were identified in the text. The analysis follows the signal from molecular input and target-induced state change through amplification, material coupling, transport, readout and interpretation, while also considering the level of evidence obtained in realistic specimens. The tables and figures throughout this review summarize these comparisons.

To make material contributions explicit, we classify each material according to its dominant analytical role: transducer (converts the molecular event into an electrical/optical signal), signal amplifier (increases the magnitude or catalytic turnover of an existing event), capture/enrichment substrate (concentrates targets or removes inhibitors), spatial organizer (controls probe orientation, spacing or local reaction geometry), or reagent-storage matrix (preserves and releases dry reagents). A material may perform more than one role, but analytical claims should identify which role is experimentally responsible for the observed improvement. This prevents attributing a lower LOD to a nanomaterial when the dominant gain actually arises from target enrichment, altered reaction kinetics or improved probe accessibility.

### 2.2. Functional Nucleic Acids as Both Receptors and Reaction Components

Functional nucleic acids are oligonucleotides whose analytical roles extend beyond the storage of sequence information. Aptamers bind molecular or cellular targets [9,10]; DNAzymes catalyze bond cleavage or generate catalytic optical signals [11,12]; structure-switching probes translate target binding into changes in distance, flexibility or accessibility [1,13,14,15]; and DNA frameworks organize probes at defined nanoscale positions on an interface [16,17,18]. In CRISPR systems, guide RNAs provide programmable recognition and activate Cas-mediated collateral cleavage that can be coupled to a reporter [19,20,21]. These categories are not mutually exclusive. An aptamer may release an HCR initiator, a DNAzyme may act as both recognizer and reporter, and a DNA tetrahedron may carry an aptamer while controlling its orientation on an electrode.

For analytical description, each functional nucleic-acid module can be defined by its input, molecular transition and output. Inputs include complementary sequences, protein ligands, metal ions, intact cells and amplification products. Hybridization, folding, cleavage, ligation, strand displacement and complex assembly are common state transitions. The resulting output may be a released initiator, an exposed G-quadruplex, a change in redox-reporter distance or an accessible material surface. This description is more informative than listing the named components of a sensing scheme without specifying how they interact.

### 2.3. Material-Assisted Transduction Is a Chemical Coupling Problem

Functional materials influence both signal generation and the chemical environment in which recognition takes place. Gold nanoparticles support thiolated probes and distance-dependent plasmonic responses [22], whereas DNA-templated metal nanoclusters provide sequence- and conformation-dependent fluorescence. Magnetic oxides can enrich targets and facilitate washing, and their intrinsic catalytic activity may also be used for color development [23,24]. Metal–organic frameworks and nanozymes combine adsorption, porosity and catalysis, but may introduce metal leaching, background activity or pore blockage [22,25]. Electrodes convert conformational changes or catalytic products into current, potential or impedance and require controlled probe density, hydration and antifouling chemistry [1,2,15,16,17]. Paper, membranes and hydrogels add capillary transport, reagent storage and convenient assay formats, together with new sources of variation related to humidity, swelling and lot-dependent flow.

**Figure 2:**
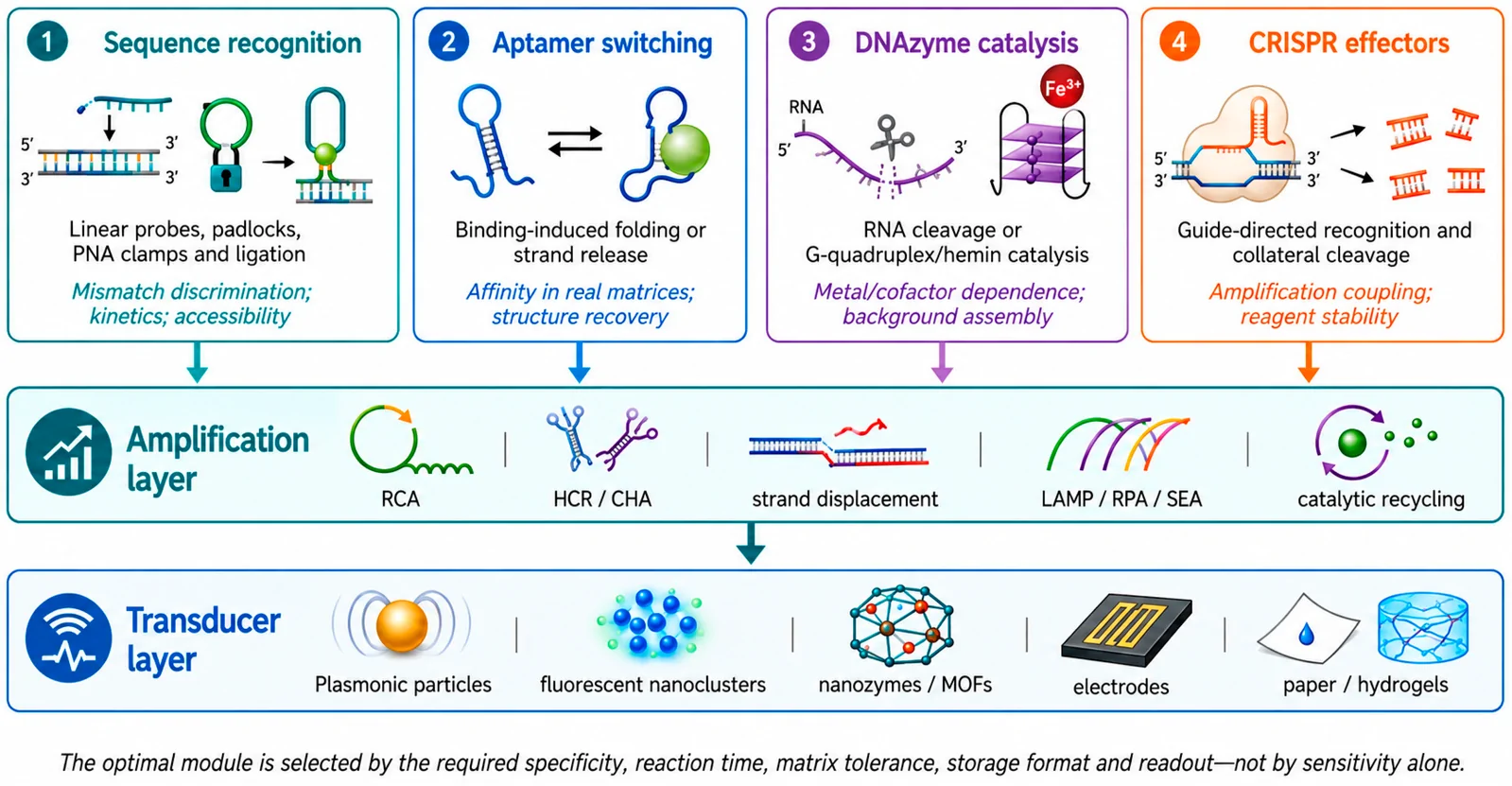
Functional nucleic-acid modules and the variables that govern their use. Recognition modules are paired with amplification and transducer layers according to specificity, reaction time, matrix tolerance, storage requirements and readout format.

The value of a material depends on the function it performs within the assay. It may increase contact between a low-abundance target and an accessible probe, separate the analyte from inhibitors, convert a weak reaction into a high-contrast optical signal, preserve dry reagents or provide an internal reference. An increase in surface area alone is not advantageous when it also increases nonspecific binding or batch variation. Figure 2 places the principal recognition modules within this broader assay sequence and shows how their selection is constrained by amplification chemistry, transducer format, reaction time, matrix tolerance and storage requirements.

The evidence framework is now treated as a multidimensional maturity map rather than a strictly sequential five-step ladder. Level 1 denotes mechanistic performance in buffer; Level 2, recovery or interference testing in spiked matrices; Level 3, testing in genuine clinical specimens against an appropriate reference method; Level 4, demonstrated reproducibility and stability across independently prepared material/device lots and relevant operators or sites; and Level 5, demonstrated clinical utility, meaning evidence that the test supports or improves the intended clinical decision or workflow rather than merely achieving diagnostic accuracy. These levels are complementary rather than strictly sequential: a study may reach Level 3 without having completed Level 4, while a technically mature device can remain below Level 5 if clinical decision benefit has not been demonstrated. Matrix recovery is not a mandatory prerequisite for every clinical study, but matrix-specific analytical characterization remains necessary for interpreting clinical performance. Manufacturing stability is therefore considered an independent translation dimension rather than equivalent to clinical comparison.

For each representative platform, we distinguish analytical performance from clinical evidence and avoid direct ranking by LOD. Quantitative context is reported where it is available in the cited study, including sample number, sensitivity/specificity, assay time and specimen type; “NR” denotes that the current source set does not provide a comparable value. Clinical studies with small cohorts are interpreted cautiously because estimated sensitivity, specificity and thresholds can have wide confidence intervals and may not generalize to a different prevalence or case mix. We emphasize performance near the intended decision threshold, sample-to-answer time, invalid/indeterminate rates, lot variation and clinical design alongside LOD. The framework is intended as a structured synthesis of evidence and reporting requirements, not as a meta-analysis or a validated scoring system.

The system-level differences among these modules are compared in Table 1, which lists their typical inputs, signal-generating transitions, main advantages, dominant risks and representative medical applications.

The risks summarized in Table 1 also determine the type and depth of evidence required before an assay can be considered relevant to medical diagnostics.

### 2.4. Evidence Hierarchy and Interpretive Limits

The evidence was considered at five levels: mechanistic performance in buffer, recovery from spiked matrices, comparison with clinical specimens, stability and reproducibility in a manufactured format, and demonstrated clinical utility. Studies limited to the first two levels were regarded as analytical proofs of principle rather than clinically validated tests. The case studies were selected to compare architectures and sources of error, not to rank diagnostic performance.

Limits of detection were not pooled or used for direct ranking because the published studies differ in specimen composition, sample preparation, reaction time, calibration, blank definition and statistical analysis. As a narrative review, the article may not represent all platforms outside the selected mechanistic categories and cannot exclude publication or citation bias. The conclusions are intended as design and reporting considerations rather than comparative estimates of clinical efficacy.

## 3. Molecular Recognition and Target-Gated Activation

### 3.1. Hybridization, Ligation and Mismatch Discrimination

Sequence complementarity provides the most direct means of recognizing nucleic-acid biomarkers, but probe design involves several trade-offs. Longer probes generally bind more strongly, yet they may tolerate mismatches and form secondary structures; shorter probes can improve mismatch discrimination but may hybridize slowly or dissociate during washing. Surface immobilization adds entropic and electrostatic constraints that are absent in solution. Probe density therefore requires optimization, since dense monolayers can restrict target access. Three-dimensional carriers, including tetrahedral DNA structures, can improve spacing and orientation compared with randomly assembled linear thiols [16,17].

Ligation introduces an additional sequence-dependent checkpoint because a circular template or reporter is formed only when adjacent probe ends are correctly aligned on the target. This approach is useful for short miRNAs and single-nucleotide variants. In one example, a T4 DNA ligase-mediated circular template specific to miRNA-378 was coupled to RCA, converting a short gastric-cancer-associated RNA into repeated products for colorimetric detection [26]. The analytical gain arose from the combination of target-specific circularization and repeated reporter generation. Ligase sequence preference, accessibility of the target ends and nonspecific circularization remain important sources of error and require mismatch controls near the clinically relevant sequence.

Peptide nucleic acid (PNA) has a charge-neutral backbone and binds complementary DNA or RNA with high affinity; it can also invade or clamp duplex structures. A saliva assay reported in 2023 combined PNA-assisted split-DNAzyme probes with recombinase-aided amplification for visual detection of *H. pylori* [27]. PNA may improve competitive hybridization and reduce undesired secondary structures, although excessive affinity can slow strand exchange or increase nonspecific retention when probe length and washing conditions are not carefully adjusted.

### 3.2. Aptamer Recognition and Structure Switching

Aptamers are obtained by iterative selection and amplification, most commonly through SELEX [9,10]. They can be chemically synthesized, reversibly folded and readily coupled to strand-displacement reactions or nanomaterials. Structure-switching aptamers convert target binding into changes in fluorescence or electron-transfer distance [13,14]. Affinity measured in selection buffer, however, often overestimates performance in serum or saliva. Divalent ions, proteins, viscosity and nonspecific adsorption alter folding equilibria, and the presentation of a target on a cell may differ substantially from that used during selection.

Measurements in real matrices confirm that high affinity in buffer does not necessarily produce an equivalent sensor response in clinical biofluids. Demek and Arroyo-Currás found that nonspecific interactions in blood plasma compressed the responses of several electrochemical aptamer-based sensors; removal of major protein and lipid fractions restored much of the sensitivity observed in buffer [28]. Chen et al. used temperature-alternated interrogation in undiluted urine and serum to compensate for sensor-to-sensor and matrix-dependent variation without a separate calibration step [29]. These studies support the inclusion of the intended matrix during selection or counter-selection and the characterization of affinity and sensor response in individual as well as pooled specimens.

Several measures can improve the performance of aptamer sensors in clinical samples. Counter-selection is more informative when it includes abundant interferents and, where feasible, the intended matrix. Calibration should focus on the clinically relevant concentration range and decision threshold. In addition, target binding is more readily distinguished from nonspecific adsorption when it produces a chemically gated event, such as strand release, split-DNAzyme assembly or steric unblocking. A turn-off DNAzyme–aptasensor for viable *C. sakazakii* illustrates the coupling of whole-cell recognition to catalytic DNA assembly and colorimetric output [30]. Although developed for a foodborne pathogen, the molecular architecture is relevant to cell-based medical diagnostics.

CRISPR diagnostics should not be treated as a single recognition/amplification category. Cas12a primarily recognizes DNA targets and, after target binding, can activate collateral cleavage of ssDNA reporters; Cas13 systems primarily recognize RNA and activate collateral RNA cleavage. Cis-cleavage refers to cleavage of the bound target or substrate, whereas trans- or collateral cleavage refers to cleavage of surrounding reporter molecules after target recognition. Cas12a activity is commonly constrained by PAM requirements, while PAM-relaxed or PAM-free architectures can shift the sequence-accessibility problem to other recognition or amplification modules. Amplification-free designs reduce primer-derived artifacts and carry-over contamination but may sacrifice sensitivity; upstream amplification increases target abundance but introduces nonspecific products and contamination risk. Targeted/cis cleavage can provide a more direct molecular event, whereas collateral cleavage converts recognition into an amplified reporter response. Accordingly, guide-dependent molecular specificity and final assay specificity must be separated; the latter also depends on sample processing, upstream amplification, reporter chemistry and background activation.

The recent CRISPR biosensor literature further illustrates these distinctions. Cas12a engineering reviews emphasize optimization of effector proteins, crRNAs, reaction buffers and diverse optical/electrochemical outputs [6], while broader reviews compare Cas9, Cas12 and Cas13 mechanisms and signal-transduction strategies [7,8]. A 2025 study coupled toehold-embedded hairpin-mediated strand displacement to Cas12a for TP53 single-nucleotide mutation detection, illustrating how an upstream molecular gate can suppress recognition-bypassing activation and improve discrimination [31]. These examples reinforce the principle that a CRISPR assay should be decomposed into target access, guide recognition, effector activation, reporter cleavage and readout rather than summarized only by its nominal specificity or LOD.

The distinction between guide-level specificity and complete assay specificity is particularly important for clinical interpretation because upstream extraction, amplification and contamination can dominate the final false-positive/false-negative pathway.

DNAzymes combine the programmability of nucleic acids with catalytic activity [11,12,32,33]. RNA-cleaving DNAzymes generally contain a catalytic core flanked by substrate-binding arms, whereas G-quadruplex/hemin systems acquire peroxidase-like activity after quadruplex folding and hemin binding. Split formats are useful because target binding can assemble otherwise inactive fragments. The same architecture can also generate background when partial complementarity or high probe concentrations promote catalyst formation in the absence of a target. Leakage is therefore better characterized from reaction kinetics than from a single endpoint blank.

G-quadruplex/hemin DNAzymes are widely used in visual assays because the reaction products can be observed without sophisticated optical equipment. Their activity is influenced by sequence context, monovalent cations, hemin concentration, surfactants and peroxide chemistry. When coupled to upstream amplification, many catalytic units can be produced from one target, as reported for *H. pylori*, *L. monocytogenes* and *C. sakazakii* [34,35,36]. Appropriate controls include target-free amplification, an amplification product without the G-quadruplex sequence, a hemin-free reaction, a catalytically inactive quadruplex mutant and a matrix blank. These controls are required to distinguish amplification leakage from the reporter background.

A recent salivary *H. pylori* assay used a pathogen-activated DNAzyme to release an HCR initiator and thereby generate an enzyme-free downstream cascade [37]. In this arrangement, pathogen recognition controls DNAzyme activation, and the released initiator subsequently drives polymer growth. Amplification is thus delayed until the target-dependent catalytic step has occurred, which reduces the opportunity for background HCR before recognition.

### 3.3. CRISPR-Coupled Recognition as a Nucleic-Acid/Material Interface

CRISPR diagnostics use guide-directed recognition to activate collateral cleavage of labeled nucleic-acid reporters [8,19,20,21]. Fluorescence, colorimetry, electrochemistry, lateral flow and surface-enhanced Raman scattering have all been used for readout. Although guide recognition is highly programmable, many assays still rely on upstream amplification and therefore remain susceptible to primer artifacts and amplicon contamination. Evaluation of a CRISPR biosensor must distinguish the contributions of amplification, guide–target recognition and Cas activation to the final signal.

Cas12a has been coupled to portable fluorescence, SERS and handheld dual-gene devices, illustrating the range of material interfaces available for CRISPR detection [8,38,39,40,41]. In another system, a trimeric G-quadruplex served as a Cas12a cis-cleavage substrate after isothermal amplification, and cleavage altered the activity of a visual DNAzyme reporter [42]. Catalytic DNA in this case replaces a conventional single fluorophore–quencher reporter and provides an additional chemical amplification step. For instrument-light use, however, sample-derived oxidants, hemin-binding compounds and lot-to-lot variation in chromogenic substrates must be assessed in the intended matrix.

## 4. Signal Propagation Networks

### 4.1. Rolling Circle Amplification: High Product Density with Interface Penalties

RCA produces a long single-stranded concatemer with repeated binding sites from a circular template [43,44]. The reaction is isothermal and can generate strong fluorescent, colorimetric or electrochemical signals. It is particularly useful when ligation provides sequence discrimination for short RNA targets or when amplification is performed in solution before product capture. The large products, however, may move slowly through porous membranes or crowded electrode layers, collapse or entangle, and limit reporter access. Product length is therefore an experimental variable that must be balanced against signal gain.

Two studies of miRNA-378 used RCA products in different optical formats. One generated repeated G-rich or reporter-binding sequences for amplified visual detection [26], whereas the other organized DNA-templated silver nanocluster probes to enhance fluorescence [45]. In the nanocluster system, RCA and fluorescence amplification were coupled because the concatemer acted as a scaffold that reorganized the probes into a higher-emission state. This arrangement avoids covalent fluorophore labeling, but the signal remains sensitive to sequence context, ionic conditions and aging of the nanoclusters. The comparative leakage and implementation trade-offs across these amplification formats are summarized in Table 2.

Across amplification formats, the dominant trade-offs can be summarized as follows. RCA provides high product density but can generate very large products that are poorly transported through porous or crowded interfaces; HCR and CHA are enzyme-free and attractive for dry formats but are sensitive to hairpin leakage, synthesis impurities and prolonged incubation; LAMP/RPA/RAA provide rapid target multiplication but can introduce primer-dependent nonspecific products and reagent-complexity burdens; catalytic enzymatic cascades can provide strong gain but add protein stability and storage constraints. Background is therefore generated by different mechanisms—spontaneous hairpin opening, nonspecific priming, incomplete ligation, catalyst assembly, reporter degradation or upstream contamination—and should be measured as a time-dependent process. Target accessibility, matrix tolerance, surface compatibility, reagent stability and manufacturability should be assessed together rather than inferred from a single endpoint signal.

RCA has also been incorporated into electrochemical assays. Chaibun et al. reported rapid electrochemical detection of SARS-CoV-2 using RCA products [46], showing that amplified nucleic acids can be converted into a portable electrical response. From a manufacturing perspective, solution-phase amplification followed by capture is often easier to characterize than surface-confined RCA because enzyme access and polymer growth are less restricted. Surface-localized RCA is most useful when spatial confinement lowers background or enables addressable multiplexing.

### 4.2. HCR, CHA and Strand-Displacement Circuits

HCR uses an initiator to open metastable hairpins and form nicked double helices [47], whereas CHA recycles an initiator through successive strand-displacement reactions [48]. Neither reaction requires a protein enzyme, which is advantageous for dry storage and low-infrastructure testing. Their main limitation is spontaneous leakage caused by imperfect hairpin metastability, synthesis impurities or temperature-dependent breathing. Since leakage increases with hairpin concentration and reaction time, endpoint optimization alone may favor conditions that raise both target signal and background.

Robust DNA circuits generally combine recognition with a separate initiation step. The target first releases or exposes an initiator, while the amplification hairpins remain kinetically inaccessible in its absence. Confinement on nanoparticles, DNA frameworks or membranes can accelerate local reactions, although excessive proximity may also increase unintended interactions. HCR has been combined with tetrahedral DNA electrodes for miRNA detection [17], magnetic nanoclusters for visual pathogen detection [49] and a pathogen-activated DNAzyme cascade [37]. In each case, performance depends on the site of initiator generation and on the ability of the polymer product to interact with the transducer.

Graphene, carbon nanotubes and MXenes are not interchangeable material classes. Graphene provides a relatively stable graphitic platform for adsorption, conductivity and quenching; carbon nanotubes provide one-dimensional conductive pathways and high aspect ratio; MXenes add hydrophilic layered surfaces and composition-dependent termination groups but may be susceptible to oxidation. Across these classes, defect density, oxidation state, surface functionalization and biomolecule adsorption can alter both signal and background. Material identity should therefore be linked to measurable interface properties rather than grouped solely by the label “carbon/2D material”.

### 4.3. Enzymatic Isothermal Amplification and Catalytic Coupling

Field-effect transistor (FET) readouts require a separate translation analysis because the effective sensing volume can be limited by Debye screening in physiological ionic strength. Signal magnitude may therefore depend strongly on ionic composition, surface functionalization, reference-gate design and transistor-to-transistor variability. Long-term operation also requires control of functionalization stability and drift. These constraints make FET performance difficult to compare directly with fluorescence or electrochemical systems unless ionic strength, gate configuration, baseline drift and device-to-device variance are reported. Accordingly, FET is treated here as a distinct transduction mode rather than as a generic member of the carbon-material category.

LAMP and recombinase-based methods reduce or eliminate the need for thermal cycling [50,51,52]. Strand exchange amplification and polymerase spiral reaction likewise generate products at a fixed temperature. Their compatibility with DNAzyme, CRISPR and lateral-flow reporters has promoted decentralized nucleic-acid testing. Nevertheless, multiple primers and high product yield can produce nonspecific amplification, and a sequence-selective downstream reporter is often required to distinguish the intended amplicon.

Catalytic reporters have been used in several ways to verify amplified products. PCR products carrying G-quadruplex sequences enabled visual detection of *H. pylori* [34]; RT-PCR products activated a G-quadruplex DNAzyme electrode response for viable *C. sakazakii* [35]; strand-exchange amplification products were quantified by RGB image analysis in saliva [53]; and Cas12a recognition separated specific from nonspecific isothermal products before a G-quadruplex visual readout [42]. In these systems, amplification supplies product mass, whereas a second sequence- or structure-dependent reaction determines whether the product generates a measurable signal.

For medical diagnostics, analytical performance is only one component of material suitability. Nanomaterial-enabled systems should also address potential toxicological concerns when residual materials can contact users or specimens, material shedding or metal/ligand leaching, disposal, physicochemical batch variation, sterilization compatibility, packaging and long-term stability. Regulatory characterization may require predefined specifications for composition, particle size/distribution, surface chemistry, impurities and release behavior. These considerations are especially important for reusable, wearable or sample-contacting devices. In an analytical review, a material should not be described as clinically ready solely because it improves signal-to-noise ratio in a laboratory assay.

For paper and lateral-flow formats, two additional failure modes deserve explicit attention. Hook effects can produce falsely low signals at very high target concentrations when excess analyte disrupts the intended capture architecture, while control-line failure can make a negative-looking strip uninterpretable. Accordingly, high-dose hook testing, control-line integrity, flow time and invalid-result rules should be part of analytical validation.

## 5. Functional Materials and Chemical Transduction

**Figure 3:**
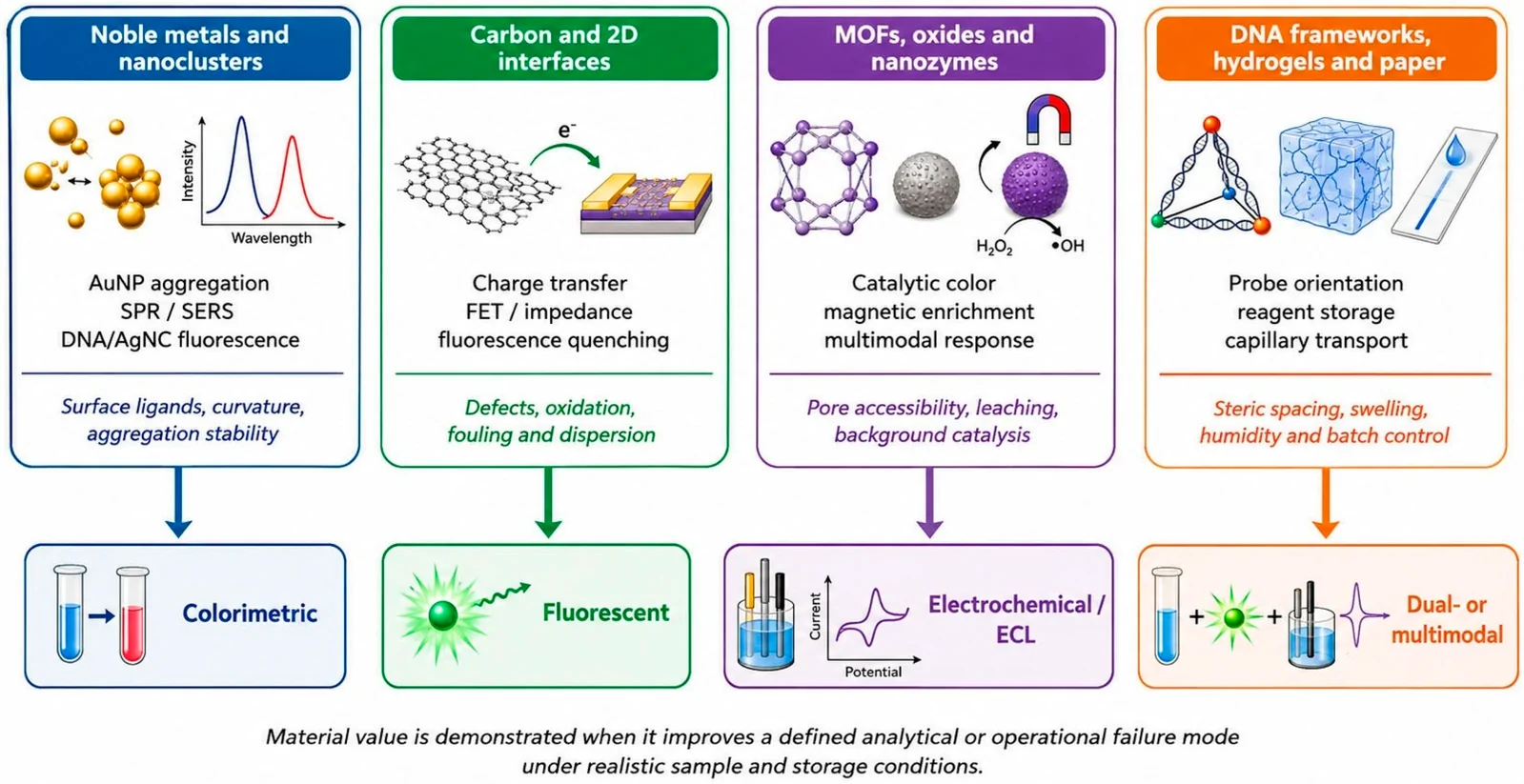
Relationships between material classes and transduction mechanisms. The map identifies the main signal mechanisms and the synthesis- or interface-dependent variables that influence performance in medical specimens.

Functional materials can be compared most clearly by considering the chemical event that each converts into a measurable output. Figure 3 maps the major material classes to their dominant optical, electrochemical and catalytic transduction mechanisms and identifies the synthesis- and interface-dependent variables that are carried into measurements in medical specimens. The following subsections examine these relationships in greater detail.

### 5.1. Noble-Metal Nanoparticles and Fluorescent Nanoclusters

Gold nanoparticles are widely used in biosensors because their optical response, surface chemistry and electron-transfer properties can be adjusted through particle synthesis and surface modification. DNA-directed assembly and distance-dependent color provided the basis for many plasmonic nucleic-acid assays [22]. In aggregation formats, signal depends on changes in interparticle coupling. Salt concentration, particle-size distribution, residual ligands and probe coverage therefore affect both sensitivity and false aggregation. On electrodes, thiol–gold chemistry provides convenient probe immobilization, but dense monolayers and ligand exchange in complex samples may reduce accessibility and stability.

Fluorescent metal nanoclusters provide a different form of coupling. Short DNA sequences can nucleate silver clusters whose emission varies with the local base environment and conformation. In an miRNA-378 assay, RCA products organized DNA/AgNC probes and increased fluorescence [45]. Nanoclusters can reduce dependence on covalent dye labeling and are smaller than many semiconductor quantum dots, but their emission is sensitive to synthesis conditions, aging and batch-to-batch photophysical variation.

### 5.2. Carbon Materials, Two-Dimensional Conductors and Electrochemical Interfaces

Graphene, carbon nanotubes, MXenes and related conductors can increase electroactive area and support redox labels, although their performance depends on defect chemistry, oxidation state and probe accessibility. Highly defective surfaces may adsorb nucleic acids strongly while producing unstable background current. Hydrophobic graphitic regions can quench fluorophores or adsorb single-stranded DNA for solution-phase switching, but proteins in clinical specimens compete for the same sites. Antifouling coatings and matrix-specific blank measurements are therefore needed when these materials are used with biofluids.

Electrochemical transduction is well suited to functional nucleic acids because target binding or folding can change reporter distance, charge transfer, interfacial capacitance or access of a soluble redox probe. Reagentless folding-based sensors may operate without washing [1,2,15,54], whereas enzymatic or DNAzyme products can provide catalytic current amplification. An RT-PCR/G-quadruplex assay coupled viable-cell RNA amplification and catalytic DNA to an electrode [35]. For clinical translation, probe density and electroactive area need to be measured or controlled, and performance should be established using independently manufactured electrode lots.

Variation among electrochemical devices reflects differences in probe loading, electroactive area, monolayer organization and interrogation method. Pellitero et al. compared interrogation metrics across independently fabricated aptamer sensors and found that peak-to-peak separation reduced temporal drift and batch variability in biological fluids [55]. Zhu et al. incorporated a second redox reporter into the self-assembled monolayer as an internal reference, improving baseline stability and reproducibility in serum and whole blood [56]. Abeykoon and White later reported single-sweep, calibration-free interrogation using continuous square-wave voltammetry [57]. These studies show that comparisons of detection limits are meaningful only after electrode-lot variation and the normalization method have been characterized.

### 5.3. MOFs, Metal Oxides, Magnetic Particles and Nanozymes

Metal–organic frameworks, porous oxides and hybrid nanozymes can combine adsorption, catalytic activity and high reporter loading. A single material may capture the target, carry nucleic-acid probes and catalyze a colorimetric reaction. This multifunctionality can make the origin of signal enhancement difficult to determine. Increased response may result from substrate concentration, catalytic oxidation or protection of the nucleic-acid probe, and these contributions require separate controls. MOF pore size, surface termination, metal leaching and batch crystallinity should be related directly to assay performance rather than reported only as material characteristics [25].

Magnetic particles are particularly useful when they reduce interference from the sample matrix. They permit target enrichment, buffer exchange and washing before signal generation. Their contribution is best established by comparing assays with and without magnetic processing in the same specimen, rather than by comparison with a particle-free assay in clean buffer. Iron oxide also has peroxidase-like activity [23,24]; this property can support integrated colorimetry but may produce background if residual particles remain after enrichment.

### 5.4. Paper, Membranes, Hydrogels and Time-Resolved Fluorescent Particles

Paper and nitrocellulose convert molecular reactions into lines or spatially separated zones. Capillary flow, low cost and visual interpretation make these substrates attractive, but transport heterogeneity, nonspecific retention and humidity-dependent reagent release can limit reproducibility. Lateral-flow development therefore involves membrane pore size, conjugate release, blocking chemistry and test-line density in addition to label brightness [58]. Smartphone readers can improve quantification and record keeping, although illumination, camera processing, and user positioning require standardization [58].

Time-resolved fluorescent microspheres provide bright labels while reducing interference from short-lived background fluorescence. A salivary *H. pylori* strip combined these particles with image analysis for visual screening and quantitative measurement [59]. The format retains a simple lateral-flow workflow and extends the reportable range when a calibrated reader is available. Reproducibility depends on a defined reading time, consistent membrane flow and label release, and prespecified roles for the visual and digital outputs. Taken together, the material classes discussed above differ not only in signal strength but also in transport, background, storage and manufacturing variation. Table 3 compares their chemical roles, compatible readouts, principal strengths, main risks and minimum validation needs. The material-dependent sources of variation summarized in Table 3 directly influence the selection and interpretation of the readout architectures discussed next.

## 6. Chemical Readout Architectures

### 6.1. Colorimetric Readout: High Accessibility, Stringent Background Requirements

Colorimetric biosensors are attractive for self-testing because they can be interpreted without specialized detectors. Color may be generated by nanoparticle aggregation, peroxidase-like oxidation, etching, particle growth or pH change. The apparent simplicity of the final readout often relies on several upstream reactions. For reliable interpretation, the hue or intensity change must remain stable during a defined reading interval and be distinguishable under common lighting conditions. Ratio-based image analysis is generally less sensitive to illumination than a single raw RGB channel, provided that the algorithm is fixed before clinical validation.

G-quadruplex/hemin DNAzymes have been incorporated into a range of visual assays [26,27,34,36,37,42]. In these systems, the major concern is not whether color can be produced, but whether target-free reactions remain below a predefined visual and statistical threshold. Autoxidation of the chromogenic substrate, hemin adsorption and the intrinsic color of the specimen must be evaluated in the complete matrix. Colorimetric assays can also be quantitative when image acquisition and analysis are standardized, as shown by RGB analysis of salivary *H. pylori* amplification products [53].

### 6.2. Fluorescence and Time-Resolved Emission

Fluorescence provides high sensitivity and allows spectral multiplexing. Functional nucleic-acid assays use fluorophore–quencher pairs, intercalating dyes, fluorescent aptamers, metal nanoclusters and enzyme-generated products. Changes in fluorescence may also arise from probe degradation, nonspecific adsorption or local polarity rather than target recognition. Time-resolved labels suppress short-lived autofluorescence, while ratiometric reporters can compensate for changes in excitation intensity and sample volume.

Two-channel assays are most informative when the signals respond to different sources of error. A fluorescence channel may provide sensitive quantification while a colorimetric channel offers visually accessible confirmation. A salivary cf-mtDNA assay combined these two modalities for copy-number measurement [60]. Interpretation requires a predefined rule for discordant results. Requiring both channels to be positive may reduce sensitivity, whereas designating one channel as a reference or confirmation signal may preserve analytical performance.

### 6.3. Electrochemical and Electrochemiluminescent Readout

Electrochemical biosensors are compatible with compact instruments, small sample volumes and printed electrodes [1,2,3]. Direct redox reporters allow reagentless measurements, whereas soluble redox probes and catalytic products often provide larger signals at the cost of additional reagents. Electrochemiluminescence combines electrical control with optical detection and can benefit from the ordered placement of probes on DNA frameworks. In all of these formats, the signal originates at an interface; nonspecific adsorption, wetting and reference-electrode instability may therefore produce changes comparable in magnitude to the molecular response.

Optimization of an electrochemical assay begins with a stable baseline in the intended matrix. Probe architecture, antifouling chemistry and the reference strategy require evaluation before additional nanomaterials are introduced. A simple printed collector with low device-to-device variation may be more useful than a complex electrode that provides a lower detection limit but poor reproducibility. Functional nucleic acids can reduce hardware requirements by combining recognition, conformational switching and amplification within the molecular layer, provided that this layer remains accessible after fabrication and storage.

For saliva-based testing, analyte concentration should be interpreted in relation to salivary flow and dilution. Where feasible, normalization to total protein, volume/time of collection, an endogenous reference or another validated internal standard should be considered. Collection time, fasting/food status, oral hygiene, hydration and device-specific recovery can otherwise introduce variability that is incorrectly attributed to the molecular sensor.

### 6.4. Multimodal Transduction and Self-Verification

Multimodal sensing can improve reliability, but the presence of two outputs does not guarantee independent confirmation. Signals derived from the same catalytic step may share a common false-positive pathway. Greater diagnostic value is obtained when the modalities rely on partly independent chemistry, such as charge-transfer measurement in one channel and catalytic color formation in another, or when one output functions as an internal reference.

Multimodal formats generally serve one of four purposes. Redundant systems measure the same event twice for confirmation; complementary systems combine a visual screen with a more sensitive instrumental measurement; ratiometric systems normalize the analytical signal to a stable reference; and logic-based systems assign different targets or controls to separate channels. The intended role and the method for resolving discordant outputs need to be specified before data analysis.

Brandsma et al. compared fluorescence and lateral-flow readouts using the same SARS-CoV-2 DETECTR chemistry. In a cohort from three hospitals, the two detector formats were fully concordant in the tested subset, and the complete CRISPR assay showed approximately 95% agreement with routine qRT-PCR across 378 clinical specimens [61]. Since the molecular recognition step, target and pre-analytical workflow were held constant, differences between the two implementations could be attributed more directly to the readout format.

## 7. Medical Diagnostic Applications

### 7.1. Pathogen Detection and Viability-Sensitive Testing

Pathogen testing must distinguish among species and, in many settings, between contamination, colonization and active infection. Nucleic-acid amplification can detect residual DNA from nonviable organisms. RNA targets, propidium monoazide treatment, metabolic activation and culture enrichment have therefore been used to provide information on viability. An RT-PCR/G-quadruplex electrochemical assay for *C. sakazakii* used RNA-associated amplification to favor detection of viable cells [35]. Although developed for food safety, the same approach is relevant to treatment monitoring and antimicrobial stewardship.

*H. pylori* is a useful example because biopsy, breath testing, stool antigen assays and serology each have specific limitations. Saliva is easy to collect but usually contains low target concentrations and substantial matrix interference. Reported salivary assays include time-resolved fluorescent lateral flow, strand-exchange amplification with RGB analysis, recombinase-aided amplification with PNA-assisted split DNAzymes and an enzyme-free DNAzyme–HCR cascade [27,37,53,59]. These formats emphasize different priorities: immunochromatography offers speed and a familiar workflow, nucleic-acid amplification improves sensitivity, PNA and split DNAzymes add sequence-dependent reporting, and HCR reduces reliance on protein enzymes in the downstream reaction.

CRISPR-based assays have also been reported for viral and bacterial genomes using fluorescence, SERS, electrochemical and lateral-flow detection [19,20,21,38,39,40,41,46]. Direct comparisons will require the same extraction procedure, specimen set and reference method for each readout. Otherwise, apparent differences in sensor performance may arise mainly from variation in target recovery rather than from the transduction chemistry.

Clinical evaluation is beginning to extend beyond small, single-center studies. A multicenter DETECTR comparison used routine specimens from three hospitals and assessed both fluorescence and lateral-flow formats [61]. The RT-LAMP–Cas12a RCSMS saliva workflow was later evaluated at two hospitals in Lima in 352 individuals and showed a reported sensitivity of 96.5% and specificity of 99.0% relative to RT-qPCR [62]. These studies provide more informative evidence because they include site-specific pre-analytical variation, operator effects, invalid results and the influence of disease prevalence on classification.

### 7.2. Cancer-Associated miRNA, cfDNA and Mitochondrial DNA

Interpretation of cfDNA and cf-mtDNA copy number depends on extraction volume, fragment size, cellular contamination and normalization. A salivary cf-mtDNA assay combined colorimetric and fluorescent readouts and reported a detection limit of 0.145 copies per microliter in artificial saliva [60]. This finding demonstrates analytical feasibility but does not establish a clinical threshold. Absolute copy-number measurements require standardized collection, centrifugation, storage and extraction, together with disease-specific reference distributions. Uncontrolled release of mitochondrial DNA from cells during handling can otherwise outweigh the benefit of a highly sensitive detector.

Complexity is a qualitative synthesis metric based on the number of oligonucleotides, enzymes, incubation/washing steps and manual interventions. “NR” means not reported or not directly comparable in the cited source. Evidence levels are assigned from the criteria defined in Section 2.4 and should not be interpreted as a validated clinical-readiness score. Food-safety and veterinary studies are explicitly separated from human medical evidence.

### 7.3. Protein, Metabolite, Extracellular-Vesicle and Cell Targets

Aptamers extend functional nucleic-acid sensing to proteins, metabolites and other non-nucleic-acid targets. Target-induced folding can change an electrochemical reporter, release an initiator for a DNA circuit or expose a catalytic domain [13,14]. In serum and other complex matrices, abundant proteins and structurally related ligands may compete with the target and reduce effective specificity. Validation therefore requires interferents at physiological concentrations and specimens from relevant disease-control groups as well as healthy donors.

Extracellular vesicles and intact cells display multiple surface ligands. Multivalent aptamer assemblies and DNA nanostructures can improve avidity, but they may also increase nonspecific membrane binding. Recognition of two independent surface markers through coincidence logic offers one means of improving specificity. DNA circuits can convert this dual recognition into a single amplified output, although the advantage must be demonstrated using single-positive controls.

### 7.4. Non-Invasive Biofluids as Design Constraints

Saliva, urine, tears and exhaled condensate simplify sample collection, but each has a composition distinct from blood. Viscosity, pH, nuclease activity, microbiota, protein content and diurnal variation differ among these biofluids. Saliva may contain mucins, food residues, oral microorganisms and cellular DNA, whereas urine varies substantially in ionic strength and pH. Assays developed in buffer may therefore require dilution, filtration, chemical lysis or magnetic cleanup before measurement.

Non-invasive testing is useful only when the selected analyte in that biofluid is biologically related to the disease state. Analytical sensitivity cannot compensate for uncertain biomarker biology. Clinical studies need to establish agreement with an accepted specimen and reference method, determine pre-analytical stability and state whether the intended use is screening, diagnosis, treatment monitoring or surveillance for recurrence.

## 8. Mechanistic Case Studies Across Integrated Architectures

**Figure 4:**
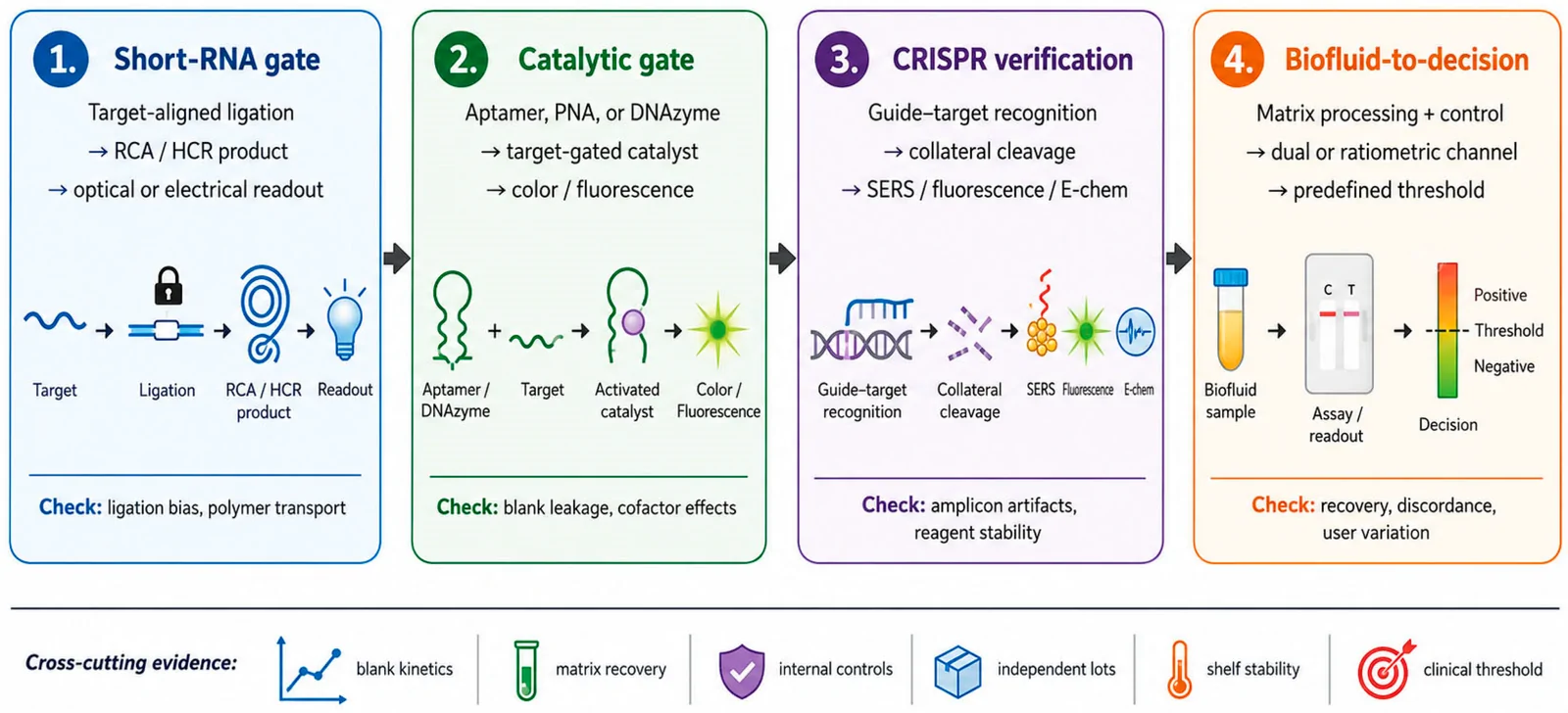
Representative integrated biosensor architectures. The examples show how molecular recognition is connected to amplification, material transduction and user-facing readout, with emphasis on the position of the specificity step, the compatibility of reaction products with the interface and the use of independent control or confirmation channels.

The preceding sections considered recognition, signal propagation, materials and readout as individual parts of a biosensor. Their analytical behavior, however, depends on how these elements are combined within a complete assay. Figure 4 brings together representative architectures to show where target specificity is imposed, how amplification products are coupled to the material interface and whether a separate control or confirmation channel is included.

### 8.1. Ligation-Gated Amplification for Short RNA Targets

Ligation-gated RCA separates sequence recognition from subsequent signal amplification. Adjacent probes must align on a short target before a circular template is formed, after which RCA generates repeated binding sites. This arrangement is suitable for miRNAs because their short length and sequence homology can limit direct hybridization assays. The miRNA-378 colorimetric and DNA/AgNC systems [26,45] demonstrate that the same upstream ligation step can support different optical readouts. Comparison of these formats requires attention to ligase bias, transport of the concatemer and sequence-dependent reporter formation in the intended specimen, rather than only to the lowest detection limit obtained in buffer.

### 8.2. Target-Gated DNAzyme and HCR Cascades

Target-gated DNAzyme cascades place catalysis after a cell-, sequence- or amplification-specific event. Aptamer-regulated DNAzyme assembly [30], PNA-assisted split-DNAzyme reporting after RAA [27] and pathogen-activated DNAzyme initiation of HCR [37] use different mechanisms to limit background before amplification. HCR-sensitized magnetic nanoclusters [49] additionally combine target enrichment with circuit amplification. Time-resolved blank measurements, inactive-catalyst controls and matrix-matched recovery are needed to distinguish target-dependent catalysis from spontaneous assembly and nonspecific amplification.

### 8.3. CRISPR–Material Coupling for Orthogonal Verification

CRISPR systems can provide a second sequence-dependent checkpoint after or alongside amplification. Cas12a and Cas13a have been connected to SERS, fluorescence, colorimetric and handheld dual-gene readouts [8,38,39,40,41]. Their main contribution is the conversion of a guide–target interaction into reporter cleavage, not simply the addition of another amplification stage. Interpretation requires separate assessment of upstream amplification artifacts, Cas activation, reporter stability and material-specific background. A dual-target or control channel is most informative when it addresses a different source of error rather than repeating the same collateral-cleavage reaction.

A practical complexity descriptor should accompany these analytical metrics. We recommend recording the number of oligonucleotides and enzymes, incubation and washing steps, total assay time, hands-on time and manual interventions. These variables determine whether an apparently sensitive architecture can realistically be deployed at the point of care. Point-of-care and decentralized testing are related but not synonymous: a point-of-care test may be performed near the patient with trained personnel and dedicated equipment, whereas decentralized testing emphasizes operation outside centralized laboratories and may impose additional requirements for self-contained workflow, robustness and user interpretation.

### 8.4. Non-Invasive and Multimodal Systems

In non-invasive and multimodal assays, sample preparation and user interpretation become part of the analytical chemistry. Salivary *H. pylori* tests based on time-resolved fluorescent lateral flow, strand-exchange amplification with RGB analysis and PNA/DNAzyme reporting [27,53,59] illustrate different balances among workflow simplicity, sequence specificity and quantitative readout. A salivary cf-mtDNA assay combined colorimetric and fluorescent signals [60], but interpretation of dual-mode results requires a predefined rule for discordance. These examples indicate that collection, internal controls, reading time and clinical thresholds must be developed together with the molecular and material components. Table 4 compares the targets, molecular processing sequences, material/readout interfaces and main analytical considerations of the representative integrated platforms discussed in this section.

Clinical variability is not synonymous with analytical variability. Age, disease stage, treatment status, circadian variation, diet, genotype and comorbidities can broaden biomarker distributions even when the assay itself is precise. A clinically weak test may therefore show excellent analytical reproducibility while retaining substantial overlap between diseased and control populations. Decision thresholds should be justified using clinically relevant distributions rather than selected solely from analytical blanks or the lowest detectable concentration.

Table 4 compares the molecular organization and analytical implications of selected platforms; it does not rank their clinical readiness. Food-safety and veterinary studies are included as mechanistic examples and should not be interpreted as evidence of performance in human diagnostic specimens.

## 9. Integrated Design Principles

Regulatory and implementation considerations should be considered early rather than appended after analytical optimization. IVD evaluation frameworks commonly separate analytical sensitivity/specificity, precision, carry-over or cross-contamination, specimen handling, clinical performance and manufacturing controls. WHO performance-evaluation procedures likewise emphasize independent verification of analytical and clinical performance, lot-to-lot variation and operational characteristics [65,66]. These principles support the review’s distinction between a high-performing laboratory chemistry and a deployable diagnostic product. Sterilization, packaging, labeling, quality systems and post-market surveillance may become relevant depending on the device format and intended use.

### 9.1. Begin with the Clinical Decision and Specimen

The intended clinical use determines the required analytical range, turnaround time and assay format. Screening tests often emphasize sensitivity and simple collection, whereas confirmatory tests place greater weight on specificity and quantitative accuracy. The specimen also determines the available volume, pretreatment options, matrix composition and target stability. These requirements are more appropriately defined before, rather than after, optimization of an assay in buffer.

An initial specification can include the intended-use population, sample type and volume, collection conditions, molecular form of the target, acceptable time to result, decision threshold, comparator method and consequences of false-positive and false-negative results. Recognition, amplification and transduction modules can then be selected in relation to these requirements.

Results from spiked samples alone do not constitute clinical validation. Clinical utility should be reserved for evidence that using the test changes or improves a clinically relevant decision, workflow or outcome for its intended population; diagnostic accuracy alone is better described as clinical validity/performance. A moderate detection limit may be sufficient when the assay reliably separates concentrations around the decision threshold, while an extreme LOD may have little clinical value if the threshold, specimen recovery or biomarker biology is unstable. Similarly, point-of-care, near-patient and decentralized testing should be described according to the actual setting, operator and infrastructure rather than treated as interchangeable terms. The minimum evidence and reporting items are summarized in Table 5.

Not all reporting items should be weighted equally. Probe identity/sequence, appropriate blank and negative controls, calibration, matrix recovery, extraction/process controls and a defined invalid-result rule are essential analytical requirements. Independent material/device lots, stability, multicenter evaluation and clinical-utility studies are translation-stage requirements whose applicability depends on the intended claim. Thus, absence of multicenter clinical validation should not be used to reject a mechanistic study, but it should prevent that study from being described as clinically validated. This staged interpretation is more appropriate than assigning equal numerical weight to every criterion.

Clinical sample size deserves explicit qualification. With only a few dozen positive and negative specimens, one or two discordant results can materially change sensitivity, specificity and the apparent decision threshold; confidence intervals may remain wide, and estimates may be strongly dependent on disease prevalence and case mix. The 352-person RCSMS study provides substantially more informative uncertainty bounds than a very small exploratory cohort, reporting sensitivity and specificity with 95% confidence intervals [62]. Clinical studies should therefore report sample counts by reference-class, confidence intervals, invalid/indeterminate rates and, where applicable, prospective versus retrospective design, blinding, and single- versus multicenter sampling.

Low-background architectures generally place the most selective molecular event before the reaction that provides the greatest gain. Examples include ligation before RCA, target-induced split-DNAzyme assembly before HCR and sequence-specific Cas recognition after isothermal amplification. By contrast, nonspecific amplification followed by a generic dye identifies product mass without independently confirming target identity. Target-dependent activation reduces this ambiguity.

Leakage from each amplification stage needs to be measured separately. In multistep assays, the contribution of individual reactions can be examined by replacing the target with a synthetic initiator, using inactive catalysts and recording blank kinetics over time. Such experiments identify the source of background and allow the cascade to be optimized without simply adding further amplification steps.

### 9.2. Match Molecular Length Scales to Material Interfaces

Functional nucleic acids provide a programmable route from molecular recognition to diagnostic readout, but their principal value is realized only when recognition, sample preparation, amplification, material coupling and interpretation operate as a coherent sample-to-answer system. The evidence reviewed here shows that molecular specificity can be lost during upstream processing, amplification or reporter generation; material properties can alter transport and background; and analytical reproducibility does not guarantee device-level reproducibility or clinical usefulness.

Three recurring design principles remain central: place the most selective molecular event before the highest-gain reaction; match amplification-product size, chemistry and transport to the material interface; and build controls that distinguish inhibition, leakage, contamination and shared false-positive pathways. These principles should be supplemented by a fourth translation requirement: quantify time, hands-on burden, complexity and independent-lot variation across the complete sample-to-answer workflow.

The relevant question is therefore not whether a platform achieves the lowest reported LOD, but whether it provides a reproducible and interpretable result in the intended specimen, around the clinically relevant decision threshold, with transparent uncertainty and an appropriate evidence level. Progress toward medical use requires matrix-specific calibration, independent material/device lots, shelf-life studies, explicit clinical-study design, regulatory characterization and predefined interpretation rules. Functional nucleic acid–material biosensors can become dependable diagnostic tools only when molecular performance is preserved through manufacturing, sample processing and clinical decision-making.

Amplification in solution followed by product capture usually provides better enzyme access and simpler kinetic analysis. Surface-localized reactions can reduce background and enable spatial multiplexing, but only when polymerases, hairpins and reporters can reach the interface. DNA frameworks offer an intermediate approach by controlling probe orientation and spacing without requiring a highly roughened surface [16,17].

### 9.3. Design Internal Controls into the Chemistry

An internal control is most informative when it experiences the same sample preparation and reaction conditions as the target. In nucleic-acid assays, an extraction or amplification control distinguishes a true negative result from reaction inhibition. Colorimetric systems may use a reference color or catalytic control to identify reagent failure. Electrochemical devices can include a reference feature or a nonresponsive redox reporter to correct for wetting and electrode variation.

In multimodal assays, at least one channel can be assigned an explicit control or confirmation function. The second signal adds the greatest value when it tests a different source of error, such as amplification success rather than target specificity, instead of reproducing the same chemistry with another detector.

### 9.4. Optimize for Information Quality, Not the Lowest Reported LOD

The limit of detection describes only one aspect of diagnostic performance. Dynamic range, precision near the decision threshold, calibration stability, matrix recovery and classification accuracy may be more important for clinical use. Very low reported limits often depend on long reaction times, high probe concentrations or extensive sample processing. Evaluation at clinically relevant concentrations and with a predefined threshold gives a more realistic measure of performance.

For quantitative assays, uncertainty estimates should include sample preparation, reagent lot and reader variation in addition to replicate wells from one batch. For qualitative assays, invalid and indeterminate results should be reported separately from negative findings.

## 10. Translation Challenges and Minimum Evidence Standards

**Figure 5:**
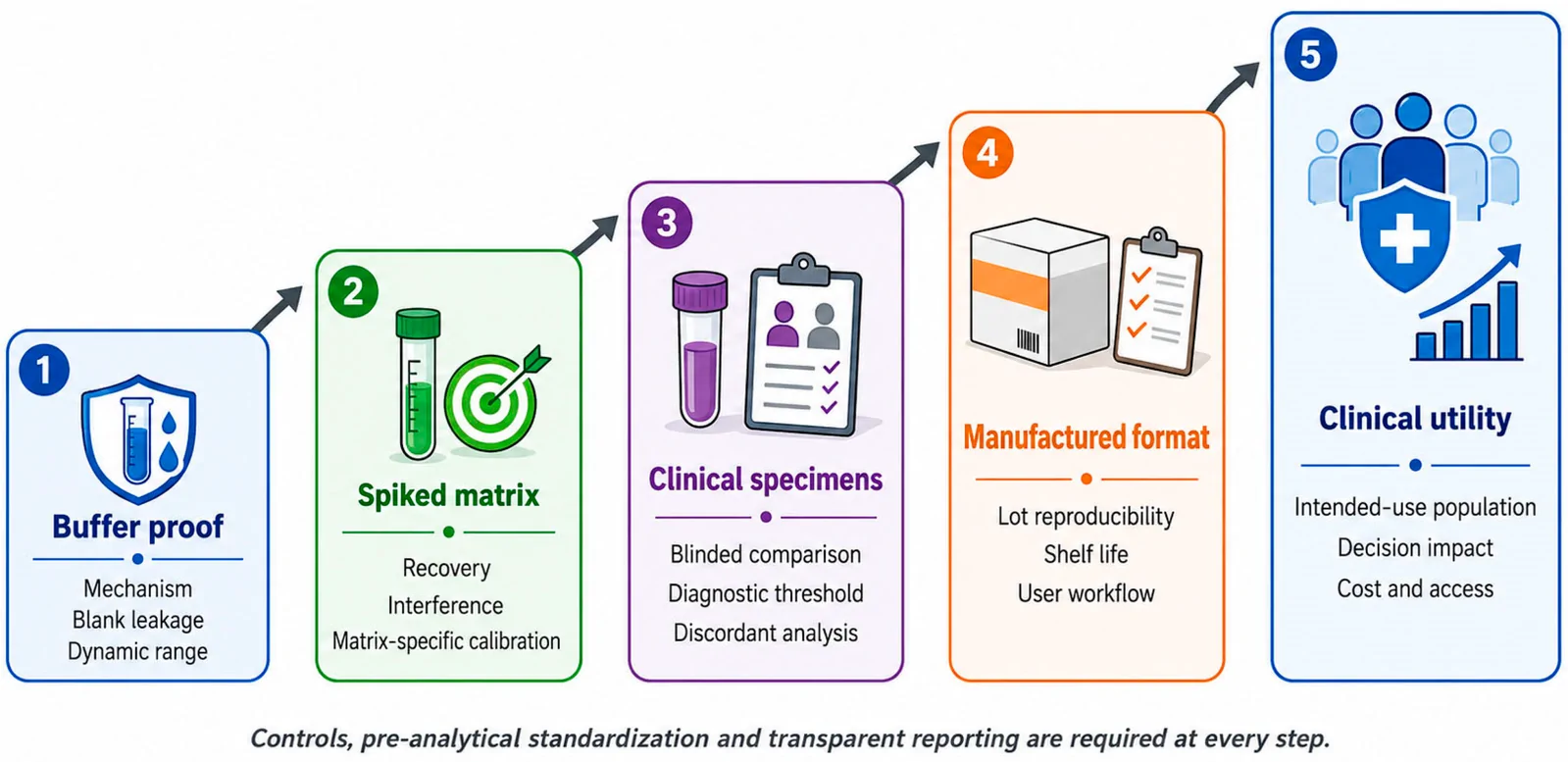
Levels of evidence for functional nucleic acid–material biosensors, from mechanistic studies in buffer to matrix recovery, clinical comparison, manufactured-format stability and clinical utility.

Translation from an analytical concept to a clinically useful test requires several distinct levels of evidence. Figure 5 organizes this progression from mechanistic studies in buffer through matrix recovery and clinical-specimen comparison to manufactured-format stability and demonstrated clinical utility. The following sections discuss the experimental and reporting requirements associated with these stages.

### 10.1. Pre-Analytical Variation and Matrix Effects

Sample collection and preparation can account for a large proportion of total assay variation. Saliva composition changes with flow rate, food intake, oral hygiene and the collection device. Plasma and serum differ in cellular contamination and in nucleic acids released during clotting. Freeze–thaw cycles and storage temperature can alter the distribution of nucleic-acid fragments. Clinical protocols therefore need to specify collection time and device, processing delay, centrifugation, stabilizers and storage limits.

Matrix evaluation should include both pooled and individual specimens because pooling can mask clinically important interference. Recovery experiments are most informative when they cover the decision range and use native or commutable materials. Dilution may reduce inhibition but also changes analyte concentration and binding equilibria; its effect must be incorporated into calibration and threshold selection.

### 10.2. Reproducibility, Lot Variation and Stability

Many academic biosensor studies report technical replicates from a single synthesis batch. Assessment for translation requires independently prepared material lots, independently manufactured device lots and more than one operator. Particle size, surface ligands, probe coverage, membrane flow and electrode baseline should be related to acceptance criteria. A functional release test is more useful than structural characterization alone when predicting the performance of a finished assay.

Studies that separate material-lot, device-fabrication and interlaboratory variation remain uncommon. Electrochemical aptamer studies have reduced sensor-to-sensor variation within individual laboratories through alternative waveform metrics, internal-reference monolayers and calibration-free interrogation [55,56,57]. The next step is evaluation of independently manufactured material and device lots across sites, with variance components reported before claims of platform-level reproducibility are made.

Accelerated aging can identify unstable components but does not replace real-time shelf-life testing. Functional nucleic acids may remain stable in the dry state while enzymes, hemin, nanoparticles or blocking reagents deteriorate. Rehydration time and humidity exposure need to be assessed in the final package. Stability claims should identify the performance measure being preserved and the corresponding acceptance limit.

### 10.3. Clinical Validation and Transparent Reporting

Clinical validation is most informative when testing is blinded and either prospective or based on a specimen set appropriate for the intended use. The study population, reference standard and specimen workflow must correspond to the proposed clinical role. Decision thresholds should be fixed before final evaluation, and discordant results should be resolved according to a prespecified procedure. Sensitivity and specificity require confidence intervals and should be reported together with invalid and indeterminate rates, quantitative signal distributions and prevalence-dependent predictive values. STARD provides established guidance for reporting diagnostic-accuracy studies [67].

The serum-vancomycin agreement study [68], the three-hospital DETECTR comparison [61] and the two-hospital RCSMS field evaluation [62] represent different approaches to validation: comparison with an automated laboratory assay, multicenter comparison with qRT-PCR and field testing of a saliva-based workflow. Reports of this type benefit from confidence intervals, predefined discordant-result analysis, invalid rates and site-specific performance.

Results from spiked samples alone do not constitute clinical validation. Clinical utility also depends on whether a test provides useful information or improves workflow relative to the current standard of care, while maintaining acceptable consequences of false-positive and false-negative results. Conversely, a moderate detection limit may be sufficient when it consistently separates clinically relevant groups. The relevant objective is reliable decision performance, not the most extreme analytical sensitivity. The minimum evidence and reporting items needed to support this objective are consolidated in Table 5.

Together, the criteria in Table 5 provide a practical reporting framework for distinguishing improvements in assay chemistry from improvements in diagnostic reliability. The remaining research needs are considered in the following section.

## 11. Future Directions

Further progress will depend on integrating recognition, amplification and material design rather than improving each component in isolation. Sequence-level co-design is one important direction: the target can be used to generate a unique initiator while downstream components remain kinetically trapped until that event occurs. Computational sequence design may help identify secondary structures and potential leakage pathways, but performance must still be verified experimentally in the intended matrix.

Reagent stabilization is equally important. Protein-free downstream reactions, including HCR and DNAzyme catalysis, may simplify dry storage, although extraction and lysis remain major practical limitations. Lyophilized master mixes, dissolvable barriers and paper or hydrogel compartments can control the order of reactions without repeated user pipetting. In these formats, materials serve not only as signal enhancers but also as reservoirs for reagent preservation and release.

Self-calibrating and multimodal assays represent another area of development. Ratiometric fluorescence, dual-electrode arrangements and color/reference zones can reduce variation caused by sample volume, illumination and device fabrication. The role of each channel should be specified in advance, with one output used for confirmation or process control when appropriate. Recent saliva-based, dual-mode and image-assisted systems illustrate this approach [53,59,60].

Multiplexing is most useful when it addresses a defined clinical uncertainty rather than simply increasing the number of detectable targets. Examples include pathogen identity together with a resistance marker, a tumor-derived nucleic acid together with a sample-quality control, or two independent surface markers on extracellular vesicles. Functional nucleic-acid circuits can implement coincidence and threshold logic, but the additional complexity is justified only when it improves classification in prospective specimens.

Manufacturability, interlaboratory comparability and validation also need to be considered early in assay development. Common reference materials, standardized matrix panels, round-robin studies and reporting of lot-level variation would improve comparison among platforms. Probe sequences, material synthesis and device geometry should be compatible with lot release, packaging, reader calibration and regulatory evaluation. Future advances are likely to depend less on achieving another record-low detection limit than on maintaining molecular specificity and assay performance from production through clinical interpretation.

## 12. Conclusions

Functional nucleic acids provide a programmable route from molecular recognition to diagnostic readout. Their main advantage lies not in any individual aptamer, DNAzyme, amplification reaction or DNA nanostructure, but in the ability to control the target-induced molecular change and its transfer to a material interface. Effective systems develop recognition, amplification, interface and readout as connected components of one assay.

The studies considered in this review identify three recurring requirements. The most selective molecular event is placed before the highest-gain reaction, the products of amplification remain physically accessible to the material interface, and the readout includes controls for inhibition, leakage and shared false-positive pathways. Ligation–RCA assays, DNAzyme–HCR cascades, CRISPR-coupled reporters and multimodal saliva tests show that these requirements can be met with different materials and in different use settings.

The relevant question is whether the complete architecture provides a reproducible and interpretable result in the intended specimen. Progress toward medical use will require matrix-specific calibration, independent material and device lots, shelf-life studies, fixed clinical thresholds and controls based on orthogonal chemistry. Functional nucleic acid–material biosensors can become dependable diagnostic tools only when molecular performance is supported by manufacturability, transparent reporting and evidence of clinical utility.

## Figures and Tables

**Table 1:** Functional nucleic-acid recognition modules and system-level design considerations.

Module	Typical Input	Signal-Generating Transition	Main Advantage	Dominant Risk	Representative Use/Evidence Status
Sequence-selective probes and ligation architectures	Complementary nucleic acids, variants and amplification products	Hybridization, ligation or strand exchange exposes/creates a reporter	High sequence programmability; single-base discrimination can be engineered	Slow surface hybridization; secondary structure; false priming	miRNA, cfDNA, pathogen genes, resistance genes; typically analytical/clinical-research use
Aptamers and structure-switching probes	Proteins, metabolites, cells and some small molecules	Binding changes conformation, releases a strand or alters surface distance	Synthetic production; reversible switching; easy chemical modification	Affinity may change in biofluids; selection buffer bias; nuclease susceptibility	Protein biomarkers, thrombin, ATP, pathogen surface targets; clinical validation varies by analyte
RNA-cleaving DNAzymes	Target-triggered assembly, metal ions or reporter substrates	Catalytic cleavage releases fluorophore, initiator or surface-bound reporter	Catalytic turnover; modular substrate arms	Cofactor dependence; leakage from partial assembly; substrate stability	Nucleic acids, cells, ions and enzyme activities; mainly analytical/mechanistic evidence
G-quadruplex/hemin DNAzymes	Amplification products or target-directed assembly	Peroxidase-like oxidation of chromogenic, chemiluminescent or electroactive substrates	Low-cost visual readout; straightforward coupling to amplification	Hemin binding and quadruplex folding are salt-sensitive; background oxidation	Pathogens, miRNA and amplified gene targets; predominantly analytical demonstrations
DNA frameworks and nanostructures	Targets presented to probes at defined geometry	Controlled spacing/orientation improves accessibility or organizes cascades	Reduced surface crowding; multiplexing; modular display	Assembly quality control; cost; structural integrity in serum	Electrochemical miRNA/protein sensing and cellular imaging; clinical utility not established by structure alone
CRISPR-associated nucleic-acid systems	Amplified or native DNA/RNA targets	Guide-directed recognition activates collateral reporter cleavage	High sequence specificity; adaptable visual/electrochemical outputs	Protein reagent storage; contamination from upstream amplification; PAM constraints	Viral, bacterial and genetic diagnostics; clinical evidence depends on specimen and validation design

**Table 2:** Comparative leakage and implementation trade-offs across amplification formats.

Amplification Format	Dominant Background/Leakage Source	Kinetic Signature	Target Accessibility	Surface Compatibility	Matrix/Reagent Sensitivity	Manufacturing Implication
RCA	Nonspecific ligation/circularization; uncontrolled polymer growth	Accumulating background with prolonged incubation	Large concatemer may restrict transport	Often easier in solution; surface RCA can crowd	Enzyme activity and ionic conditions	Template/enzyme lots; product-length QC
HCR	Hairpin breathing, synthesis impurities, spontaneous initiator-independent opening	Background increases with hairpin concentration/time	Polymer size can limit reporter access	Compatible with surfaces but crowding matters	Salt/temperature and oligo quality	Hairpin purity and lot QC
CHA	Toehold leakage and unintended strand displacement	Gradual baseline increase; sensitive to toehold design	Usually smaller intermediates than RCA/HCR	Generally surface-compatible if access preserved	Sequence/temperature dependent	Oligo purity and sequence QC
LAMP/RPA/RAA	Nonspecific priming, primer-dimers, carry-over contamination	Rapid target and background growth; endpoint may mask origin	Amplicons generally accessible but heterogeneous	Downstream capture/reporting often needed	Enzymes, primers, inhibitors	Primer/enzyme lots; contamination control

**Table 3:** Functional materials, transduction mechanisms and the principal sources of analytical variation.

Material Class	Chemical Role	Compatible Readouts	Strength	Main Risk	Principal Batch-to-Batch Variation/Minimum Validation
Au nanoparticles/nanostructured Au	Plasmonic coupling, SERS, electron transfer, thiol immobilization	Colorimetric, SERS, electrochemical	Strong signal; established conjugation chemistry	Aggregation instability; ligand contamination; probe crowding	Particle-size distribution, zeta potential, probe density, matrix-induced aggregation; independent lots and functional release testing where translation is claimed
DNA-templated metal nanoclusters	Sequence- and conformation-dependent emission	Fluorescence	Small labels; label-free synthesis; switchable emission	Batch photophysics; aging; salt sensitivity	Emission spectrum, quantum yield proxy, aging and sequence controls; independent lots and functional release testing where translation is claimed
Carbon and 2D materials	Adsorption, quenching, charge transfer, high-area conduction	Fluorescence, impedance, voltammetry, FET	Fast electrical readout; flexible/printed integration	Fouling; oxidation drift; poorly controlled defects	Raman/XPS linked to blank current, probe accessibility and matrix recovery; independent lots and functional release testing where translation is claimed
MOFs/nanozymes/metal oxides	Porous loading, catalytic oxidation, magnetic enrichment	Colorimetric, electrochemical, photothermal, multimodal	Capture and transduction in one material	Leaching; pore blocking; catalytic background	Batch crystallinity, metal release, catalytic blank, recovery after enrichment; independent lots and functional release testing where translation is claimed
DNA frameworks	Defined probe spacing and orientation	Electrochemical, fluorescence, ECL	Reduced crowding; modular multiplexing	Assembly QC; cost; nuclease stability	Gel/AFM assembly evidence, surface coverage, serum stability; independent lots and functional release testing where translation is claimed
Paper/membranes/hydrogels	Capillary transport, compartmentalization, reagent storage	Visual, lateral flow, fluorescence	Low-cost workflow; instrument-light use	Humidity and flow variation; nonspecific retention	Flow time, line CV, humidity challenge, accelerated and real-time stability; independent lots and functional release testing where translation is claimed

**Table 4:** Representative integrated architectures and the main analytical consideration illustrated by each platform.

Year	Target/Application	Context	Recognition and Signal Processing	Readout/Material Interface	Evidence Level	Quantitative Context	Clinical Study Design	Total Time/Hands-On	Complexity	Transferable Lesson/Ref.
2011	Small molecules	Mechanistic	Ligation-triggered DNAzyme cascade	Fluorescence	Level 1	NR	Not clinical	NR/NR	NR	Ligation gate before catalytic amplification [63]
2014	miRNA	Mechanistic	Tetrahedral DNA probe + HCR	Electrochemical electrode	Level 1	NR	Not clinical	NR/NR	NR	Probe orientation matched to polymer growth [17]
2019	miRNA-378	Analytical	Target-specific ligation + RCA	Colorimetric	Level 1–2	NR	Not clinical	NR/NR	NR	Short-RNA specificity gate before RCA [26]
2020	Viable *C. sakazakii*	Food-safety model	Aptamer-regulated split/turn-off DNAzyme	Colorimetric	Level 1–2	NR	Not clinical; food-safety	NR/NR	NR	Mechanistic architecture; no medical readiness claim [30]
2021	SARS-CoV-2 RNA	Analytical/portable	Target amplification + RCA	Electrochemical	Level 1–2	NR	Not clinical validation in this comparison	NR/NR	NR	Solution amplification can simplify capture [46]
2022	Dual viral genes	Analytical/portable	Orthogonal Cas12a/Cas13a trans-cleavage	Handheld fluorescence	Level 1–2	NR	Not clinical validation in this comparison	NR/NR	High	Orthogonal effectors enable sequence verification [41]
2023	*H. pylori* in saliva	Clinical-specimen oriented	RAA + PNA-assisted split DNAzyme	Colorimetric	Level 2–3	NR	Saliva; clinical design NR in current source set	NR/NR	High	Sequence-selective catalytic gate after amplification [27]
2023	African swine fever virus	Veterinary model	HCR-sensitized magnetic nanoclusters	Visual + magnetic enrichment	Level 1–2	NR	Not human clinical; veterinary	NR/NR	High	Enrichment + enzyme-free amplification; not medical evidence [49]
2024	African swine fever virus genes	Veterinary model	CRISPR/Cas12a	SERS nanostructure	Level 1–2	NR	Not human clinical; veterinary	NR/NR	High	Guide recognition + Raman readout; not medical evidence [38]
2025	Salivary cf-mtDNA	Human biofluid/analytical	Amplification-coupled dual reporter	Colorimetric/fluorescent	Level 2–3	0.145 copies/µL in artificial saliva	Clinical utility not established	NR/NR	High	Dual modes require predefined discordance rules [60]
2021	SARS-CoV-2	Clinical	DETECTR chemistry; fluorescence vs lateral flow	Fluorescence/lateral flow	Level 3	~95% agreement with qRT-PCR; *n* = 378	Three hospitals; multicenter comparison [61]	NR/NR	Moderate	Same molecular chemistry enables readout comparison [61]
2024	SARS-CoV-2 in saliva	Clinical	RT-LAMP + Cas12a RCSMS	Lateral flow	Level 3	*n* = 352; sensitivity 96.5%, specificity 99.0%; 40 min	Prospective cross-sectional; 2 hospitals; consecutive non-probability sampling [62]	40 min/NR	Moderate–high	Pre-analytical and operator effects are part of performance [62]
2025	Serum NAP2	Clinical research	Electrochemical aptamer sensor	Electrochemical	Level 3	10 µL serum; concentration in 5 min; AUC 0.95 for stage I [64]	Human serum; clinical comparison; exact cohort size NR in current manuscript	5 min/NR	Low–moderate	Specificity to NAP2 vs precursor and rapid quantitative readout [64]

**Table 5:** Minimum evidence and reporting items for functional nucleic acid–material biosensors intended for medical diagnostics.

Domain/Priority	Minimum Evidence	Why It Is Necessary
Essential	Target sequence/epitope, probe sequence, predicted structure, affinity or melting behavior, mismatch and interferent panel	Demonstrates that specificity arises from the intended molecular gate
Essential	Reaction time course, leakage rate, inactive-component controls, initiator titration, contamination controls	Separates true amplification from background growth
Essential	Lot identity, size/chemistry, probe density, accessibility, blank signal, matrix fouling	Links materials characterization to assay function
Essential	Collection device, timing, processing, storage, extraction efficiency, lysis/enrichment/inhibitor removal, invalid-rate definition, matrix normalization	Makes sample-to-answer performance reproducible and separates pre-analytical from molecular failure
Essential	Collection device, timing, processing, storage, extraction efficiency, invalid-rate definition	Makes results reproducible across sites and populations
Essential for translation	Intended-use population, blinded comparison where feasible, reference standard, prespecified threshold, confidence intervals, discordant analysis, prospective/retrospective and single-/multicenter designation	Establishes diagnostic performance and its uncertainty; prevents small-cohort overinterpretation
Essential for clinical claims	Hands-on steps, total time, hands-on time, reader calibration, lighting/color-vision effects, hook-effect testing, control-line integrity, invalid/indeterminate outputs	Ensures that a chemical signal becomes a reproducible user decision
Essential for user-facing testing	Hands-on steps, time to result, reader calibration, color-vision/lighting effects, invalid and indeterminate outputs	Ensures that chemical signals become reliable user decisions

## Data Availability

No new data were created or analyzed in this study. Data sharing is not applicable to this article.

## Supplementary Materials

The following supporting information can be downloaded at: https://www.mdpi.com/article/10.3390/molecules31183358/s1, Figure S1: Transparent literature-identification and selection logic for this critical narrative review. The flow records the databases/source classes, search concepts, citation tracking, inclusion/exclusion rationale and the limitation that retrospective record counts were not prospectively captured.

## Abbreviations

AgNC, silver nanocluster; AuNP, gold nanoparticle; cfDNA, cell-free DNA; cf-mtDNA, cell-free mitochondrial DNA; CHA, catalytic hairpin assembly; CRISPR, clustered regularly interspaced short palindromic repeats; ECL, electrochemiluminescence; FET, field-effect transistor; HCR, hybridization chain reaction; LAMP, loop-mediated isothermal amplification; LOD, limit of detection; LOQ, limit of quantification; MOF, metal–organic framework; PCR, polymerase chain reaction; PNA, peptide nucleic acid; RAA, recombinase-aided amplification; RCA, rolling circle amplification; RPA, recombinase polymerase amplification; RT-PCR, reverse-transcription polymerase chain reaction; SERS, surface-enhanced Raman scattering; TRFM, time-resolved fluorescent microsphere.
