# Supplementary material for: From Molecular Recognition to Clinical Readout: Design Principles for Functional Nucleic Acid–Material Biosensors in Medical Diagnostics
Source: Molecules. 2026 Sep 21;31(18):3358. doi: 10.3390/molecules31183358 (PMC13609665; doi:10.3390/molecules31183358)
Supplement: Supplementary file 1 [file molecules-31-03358-s001.zip › molecules-4518281-supplementary.pdf]

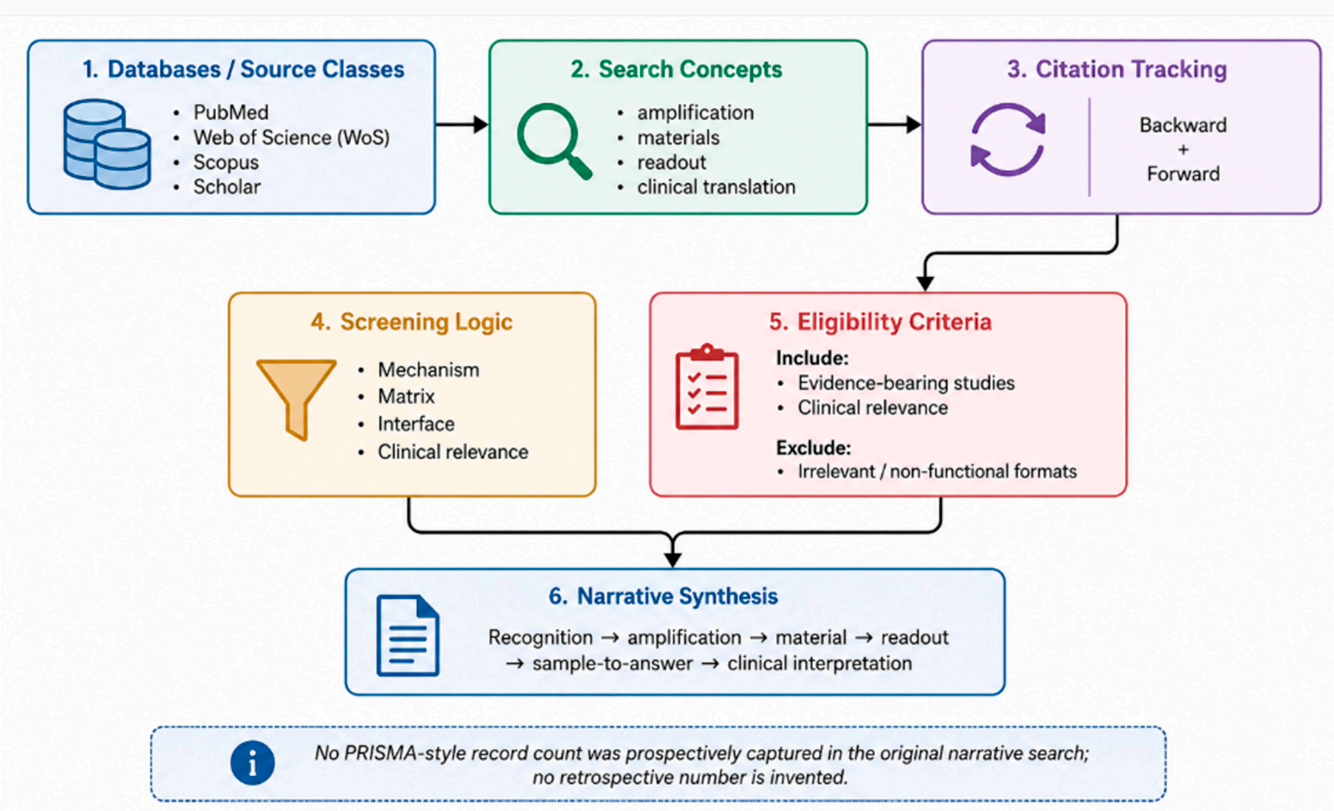

Figure S1. Transparent literature-identification and selection logic for this critical narrative review. The flow records the databases/source classes, search concepts, citation tracking, inclusion/exclusion rationale and the limitation that retrospective record counts were not prospectively captured.
